# Agile Health Care Analytics: Enabling Real-Time Disease Surveillance With a Computational Health Platform

**DOI:** 10.2196/18707

**Published:** 2020-05-28

**Authors:** Wade L Schulz, Thomas J S Durant, Charles J Torre Jr, Allen L Hsiao, Harlan M Krumholz

**Affiliations:** 1 Department of Laboratory Medicine Yale School of Medicine New Haven, CT United States; 2 Center for Outcomes Research & Evaluation Yale New Haven Hospital New Haven, CT United States; 3 Information Technology Services Yale New Haven Health New Haven, CT United States; 4 Department of Pediatrics Yale School of Medicine New Haven, CT United States; 5 Section of Cardiovascular Medicine Department of Internal Medicine Yale School of Medicine New Haven, CT United States; 6 Department of Health Policy and Management Yale School of Public Health New Haven, CT United States

**Keywords:** real-time analytics, real-world data, disease surveillance, computational health, surveillance, public health, COVID-19, outbreak, health information technology, HIT, interface, monitoring, pandemic

## Abstract

The ongoing coronavirus disease outbreak demonstrates the need for novel applications of real-time data to produce timely information about incident cases. Using health information technology (HIT) and real-world data, we sought to produce an interface that could, in near real time, identify patients presenting with suspected respiratory tract infection and enable monitoring of test results related to specific pathogens, including severe acute respiratory syndrome coronavirus 2. This tool was built upon our computational health platform, which provides access to near real-time data from disparate HIT sources across our health system. This combination of technology allowed us to rapidly prototype, iterate, and deploy a platform to support a cohesive organizational response to a rapidly evolving outbreak. Platforms that allow for agile analytics are needed to keep pace with evolving needs within the health care system.

The ongoing coronavirus disease (COVID-19) outbreak demonstrates the need for novel applications of real-time data to produce timely information about incident cases [1]. Using health information technology (HIT) and real-world data (RWD), we sought to produce an interface that could in near real time, identify patients presenting with suspected respiratory tract infection and enable monitoring of test results related to specific pathogens, including severe acute respiratory syndrome coronavirus 2 (SARS-CoV-2).

This tool was built upon our computational health platform (CHP), which provides a data integration and analysis platform across our health system [2]. The platform enriches clinical data with geolocation information and real-time data from our enterprise integration engine. Data within our clinical data warehouses are transformed into the Observational Medical Outcomes Partnership (OMOP) common data model [3] and merged with data from the integration engine to support real-time analytics and promote the capacity for rapid collaboration. We developed an interactive dashboard to summarize these data and present them in a format suitable for analysis (Figure 1).

**Figure 1:**
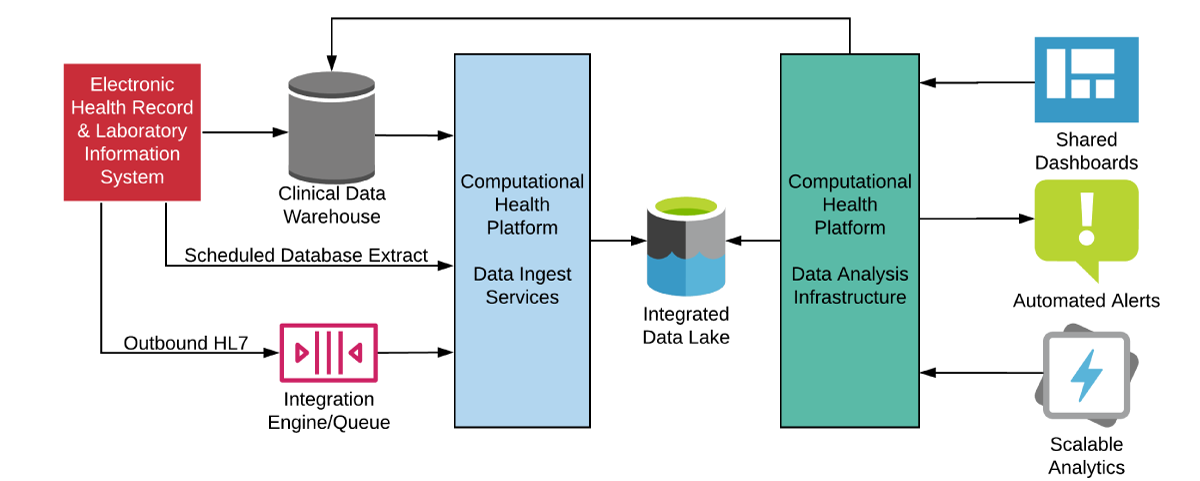
The architecture of data flows within the computational health platform with key integration points that allow for real-time data access and agile analytics. HL7: Health Level 7.

Factors impeding data access included limitations in electronic health record (EHR) interfaces and manual reporting workflows. After moving to an integrated EHR and laboratory information system, our Health Level 7 order and results feed became limited to results, which prevented access to other essential data such as order information, requiring us to develop a method to extract data from the underlying clinical database. The second bottleneck was the manual workflow in reporting COVID-19 results from the state reference laboratory, which initially relied on faxed paper results.

This real-time platform provides several benefits for managing a cohesive organizational response to a rapidly evolving outbreak. Trends can be tracked, issues with individual patients can be identified, hot spots can be determined, and new patients can be automatically reported for contact tracing. Delays in testing and resulting can also be identified across an integrated health care delivery system to address potential barriers to care delivery. Since these data are integrated with our OMOP data repository, downstream apps and timely observational research are also supported.

From March 1, 2020, through May 14, 2020, the tools described here were used to follow over 40,000 patients tested for COVID-19 across our health system, with information regarding admissions, intensive care unit capacity, and ventilator use for over 9600 patients with positive SARS-CoV-2 results. Dashboards were created to track laboratory testing volumes, turnaround times, and outstanding tests. The platform was also used to create a clinical COVID-19 data registry to support operational analytics, quality improvement initiatives, and biomedical research across the organization, and to create clinical predictive models [4].

With the rapid increase in COVID-19 and associated morbidity and mortality, there is a noted urgency to leverage available resources to identify risk factors and possible treatments, and track outcomes. For this, high-quality data are needed to generate evidence [5]. Although randomized controlled trials are being implemented internationally, they have several limitations, including typically long time periods from initiation to results. As such, other methods are needed to provide short-term information that can be used to guide the clinical and organizational response to a rapidly evolving pandemic. RWD has the potential to offer important insights into the management of COVID-19 and for the development of clinical predictive models that can be used in frontline care. Many investigators have begun to take advantage of RWD for COVID-19 research, but, although the information generated from RWD sources can be valuable, ensuring high-quality analysis requires new methodologic approaches, significant data validation, and careful interpretation of results.

Platforms that allow for agile analytics are needed to keep pace with evolving needs within the health care system. By leveraging our existing CHP, the initial tool was created and sourced with near real-time data in approximately 1 day of development effort from one developer and one clinical informaticist. These systems require an integrated team of HIT experts, clinical informaticists, health care providers, and administrative support to ensure that high-quality data can be efficiently disseminated across the enterprise. Such systems can be used to generate the RWD needed to drive evidence generation, especially with a rapidly evolving pandemic, but care is needed to ensure high-quality data are produced and that results are correctly interpreted given their observational nature.

## Abbreviations

CHP: computational health platform
COVID-19: coronavirus disease
EHR: electronic health record
HIT: health information technology
OMOP: Observational Medical Outcomes Partnership
RWD: real-world data
SARS-CoV-2: severe acute respiratory syndrome coronavirus 2
